# Mesenchymal stem cells to treat diabetic neuropathy: a long and strenuous way from bench to the clinic

**DOI:** 10.1038/cddiscovery.2016.55

**Published:** 2016-07-11

**Authors:** J Y Zhou, Z Zhang, G S Qian

**Affiliations:** 1 National Drug Clinical Trial Institution, Xinqiao Hospital, Third Military Medical University, Chongqing 400037, China; 2 Institute of Respiratory Diseases, Xinqiao Hospital, Third Military Medical University, Chongqing, 400037, China

## Abstract

As one of the most common complications of diabetes, diabetic neuropathy often causes foot ulcers and even limb amputations. Inspite of continuous development in antidiabetic drugs, there is still no efficient therapy to cure diabetic neuropathy. Diabetic neuropathy shows declined vascularity in peripheral nerves and lack of angiogenic and neurotrophic factors. Mesenchymal stem cells (MSCs) have been indicated as a novel emerging regenerative therapy for diabetic neuropathy because of their multipotency. We will briefly review the pathogenesis of diabetic neuropathy, characteristic of MSCs, effects of MSC therapies for diabetic neuropathy and its related mechanisms. In order to treat diabetic neuropathy, neurotrophic or angiogenic factors in the form of protein or gene therapy are delivered to diabetic neuropathy, but therapeutic efficiencies are very modest if not ineffective. MSC treatment reverses manifestations of diabetic neuropathy. MSCs have an important role to repair tissue and to lower blood glucose level. MSCs even paracrinely secrete neurotrophic factors, angiogenic factors, cytokines, and immunomodulatory substances to ameliorate diabetic neuropathy. There are still several challenges in the clinical translation of MSC therapy, such as safety, optimal dose of administration, optimal mode of cell delivery, issues of MSC heterogeneity, clinically meaningful engraftment, autologous or allogeneic approach, challenges with cell manufacture, and further mechanisms.

## Facts

- Diabetic neuropathy (DN) often causes foot ulcers and even limb amputations, without efficient therapy.
- DN shows declined vascularity in peripheral nerves and lack of angiogenic and neurotrophic factors.
- Preclinical and clinical studies indicate that mesenchymal stem cell (MSC) therapy restores manifestations of DN.

## Open questions

- What is the exact molecular mechanism of MSCs on DN?
- Are there any molecules secreted by MSCs to protect bone marrow nerve and to maintain bone marrow homeostasis?
- Which challenges would be most difficult in the clinical translation of MSC therapy?

## Introduction

DN is one of the most frequent complications of diabetes, 66% for type 1 diabetes and 59% for type 2 diabetes.^1^ The pathophysiology of DN is complicated and not fully elucidated that involves both vascular and neural components. DN is a systemic and progressive disorder and its manifestations need many years to develop. Intervention with tight blood glucose control and treatment with aldose reductase inhibitor or *α*-lipoic acid successfully inhibit the progression of DN,^2,3^ but no established curable treatment is available during the progressive stage. During the past decades, one of the innovative preclinical study has applied gene therapy or MSC therapy to DN in animal models,^4^ but gene therapy shows weak result or is ineffective.

MSCs have been believed as a promising regenerative therapy for DN because of their multipotency and their paracrine secretion of angiogenic factors and neurotrophic factors. Umbilical cord blood *ex vivo* expanded CD34 and umbilical cord matrix MSCs were well tolerated without adverse effects in a 29-year-old male.^5^ MSC therapies offer more benefits than other cell-based therapies. Practically, as the safety of autologous bone marrow-derived MSCs (BMSCs) have been documented by variety of clinical trials,^6^ it is highly recommended to use this strategy in a pilot clinical trial for those who are severely affected by DN. In this review, we will briefly summarize the pathogenetic mechanisms, effects of MSC treatment, and challenges from bench to bedside studies of MSCs on DN.

## Diabetic neuropathy

**Figure 1:**
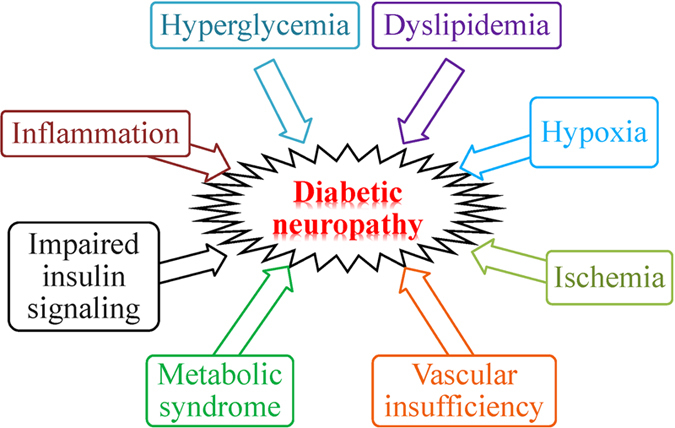
Pathogenesis of diabetic neuropathy.

DN is characterized with progressive neuronal loss, demyelination, and damaged nerve regeneration with ultimately dysfunction of nerve fibers impairing both the autonomic and somatic divisions of the nervous system.^7^ The pathogenesis of DN is complex but the same as other complications, hyperglycemia exacerbates its development. Hyperglycemia damages neurons, Schwann cells, and endothelial cells of the vasa nervorum in the peripheral nerves. Hyperglycemia results in oxidative stress, reactive oxygen species generation, and advance glycation end product production, which leads to impairment in sensory, motor, and autonomic nerve.^8^ Several factors involve in the development and progression of DN (Figure 1).^7–11^

### Role of neurotrophic factors in pathogenesis

Except the classical major pathophysiological role of neurotrophic factors and vascular supply in DN, the two widely considered downstream consequences of the cellular mechanisms are the loss of neurotrophic support and ischemic hypoxia. Direct cellular contact is not necessary to provide neuroprotection.^12–14^ Critical in providing a protective microenvironment, neurotrophic factors are growth factors known to promote neuron development and survival. They also maintain functional integrity, promote regeneration, regulate neuronal plasticity, and aid in the repairing of damaged nerves.^15^ The various protective types of neurotrophic factors affect different cell populations within the peripheral and central nervous system.

Deficiency of these neurotrophic factors can cause development of DN.^16^ Diabetes reduces brain-derived nerve factor (BDNF), nerve growth factor (NGF), and neurotrophin 3 in peripheral nerves by limiting anterograde and retrograde axonal transport. Intrathecal delivery of NGF or neurotrophin 3 improves myelinated fiber innervation in the dermal footpad of diabetic mice, and thus lack of neurotrophic support affect fiber morphology. Neurotrophic factors may regulate angiogenesis. BDNF is an essential factor in maintaining cardiac vessel-wall stability during development.^17^ NGF stimulates angiogenesis indirectly by increasing the expression of (vascular endothelial growth factor (VEGF) and directly by promoting vascular cell growth. Both neurotrophin 3 (through binding to TrkC) and leukemia-inhibitory factor serve as inhibitors of the growth of some endothelial cells.

### Role of angiogenic factors in pathogenesis

Many representative growth factors VEGF, insulin-like growth factor, NGF, BDNF, and fibroblast growth factor-2 (FGF2, also known as bFGF) have dual effects of being both neurotrophic and angiogenic. These growth factor levels are decreased in diabetic animals and are associated with neural function.^18,19^

VEGF, a major angiogenic factor, is a potent selective mitogenic cytokine for endothelial cells. VEGF enhances migration and proliferation of Schwann cells and promotes axonal outgrowth and survival of both the neurons and Schwann cells of superior cervical ganglia and dorsal root ganglia. Insulin-like growth factors promote neurite outgrowth of neuroblastoma cells, accelerate regeneration of sensory and motor nerves, and stimulate Schwann cell mitogenesis and myelination. NGF provides neuroprotective, repair functions, and directly induces angiogenesis via promoting survival and differentiation of sensory and sympathetic neurons.^20^ NGF homozygous knockout mice do not develop proper sympathetic neurons or small neural crest-derived sensory neurons.

## Mesenchymal Stem Cells

### MSC classification

MSCs have the capacity of self-renewal and the potential to differentiate into multiple cell types such as adipocytes, chondrocytes, and osteoblasts, myocytes, and neurons.^21–24^ MSCs can be derived from bone marrow, adipose tissue, nervous tissue, amniotic fluid, umbilical cord, placenta, menstrual blood, and dental pulps.^25–31^ BMSCs and adipose tissue-derived MSCs are representativeof this.^32,33^

MSCs are a subset of cells that express on their surface CD54/CD102, CD166, and CD49 as well as CD73 and CD90. They also express CD44 and CD105, whereas they do not express CD34, CD14, CD45, CD11a/LFA-1, and CD31, which are surface markers of hematopoietic cells and/or endothelial cells.^34,35^ Although their differentiation capacity is less than other cell types such as embryonic stem cells or induced pluripotent stem cells, MSCs migrate and home to injured sites, acting both by regenerating tissues and by secreting trophic factors and paracrine mediators. MSCs have remarkable immunosuppressive properties secreting cytokines and immunomodulatory substances.^36–41^

### MSCs secret neurotrophic and angiogenic factors

Delivering neurotrophic or angiogenic factors in the form of protein or gene for therapy have no significant effect. BMSCs are effective for reversing various manifestations of experimental DN.^7^ MSCs secrete various cytokines with angiogenic and neurosupportive effects. MSCs reside in the BM stromal fraction, which provides the cellular microenvironment supporting hematopoiesis. MSCs are adherent and expandable in culture, which makes it relatively easy to obtain a sufficient number of cells for MSC therapy.

Human MSCs (hMSCs) produce 84 trophic factors in conditioned medium and/or cell lysates *versus* basal medium.^42^ Human umbilical cord blood MSC treatment partially restore the neuronal degeneration and nerve function of femoral nerve.^43^ Human umbilical cord-derived MSCs secrete VEGF, glial cell line-derived neurotrophic factor (GDNF), and BDNF. Secretion of neurotrophic factors is demonstrated before, during, and after neuronal differentiation. Human umbilical cord-derived MSCs and BMSCs both had measurable amounts of secreted neurotrophic factors. But *in vivo* tests did not confirm the secretion of neurotrophic factors and the antiapoptotic effects seen *in vitro*.^44^

Dental pulp stem cells express various neurotrophic factors, including BDNF, NGF, and GDNF.^12^ The transplantation of cryopreserved dental pulp stem cells attenuate sciatic nerve blood flow and sciatic nerve conduction velocity the same as freshly isolated dental pulp stem cells.^45^ Similarly, adipose-derived stem cells differentiated to glial-like cells also express a range of neurotrophic factors, namely NGF, BDNF, GDNF, and neurotrophin 4. MSC transplantion into an animal model of nerve injury show antiapoptosis in the dorsal root ganglia.^46^ Adipose tissue-derived stem cells isolated from the ischemic limb of diabetic patients have less potent phenotypically and functionally compared with control normal counterparts without signs of limb ischemia.^47^

### Neuroprotective and neuroregenerative mechanisms

The secretion of neurotrophic factors by stem cells provides neuroprotection and neuroregenerative effects. When transplanted into an animal model of Parkinson’s disease, hMSCs support sustained endogenous proliferation and maturation of cells in the subventricular zone of rats. Additionally, hMSCs exert a neuroprotective effect, decreasing the loss of dopaminergic neurons and increasing the levels of dopamine in the animal models of Parkinson’s disease.^42,48^ These effects are possibly accomplished through decreased caspase-3 activity. hMSC-treated mice have a lower removal times than that injected with proteasome inhibitors and no hMSC transplantation.^48^ Transplanted hMSCs did not differentiate into a neural phenotype^42^ and protected against Purkinje cell loss.^49^ These studies demonstrate that MSCs not only protect against nerve damage but also help regenerate damaged nerves and restore them to their preinjured state.

The secretion of neurotrophic factors by different populations of stem cells suggests that no matter the source MSCs have the ability to decrease and ameliorate the negative effects on injured nerve fibers, improving the function of the injured nerve. The release of key neurotrophic factors, along with the neuroprotective and neuroregenerative effects of stem cells, make them ideal candidates for arresting and possibly reversing the incapacitating effects of DN.

## Mechanisms of MSC treatment for DN

**Figure 2:**
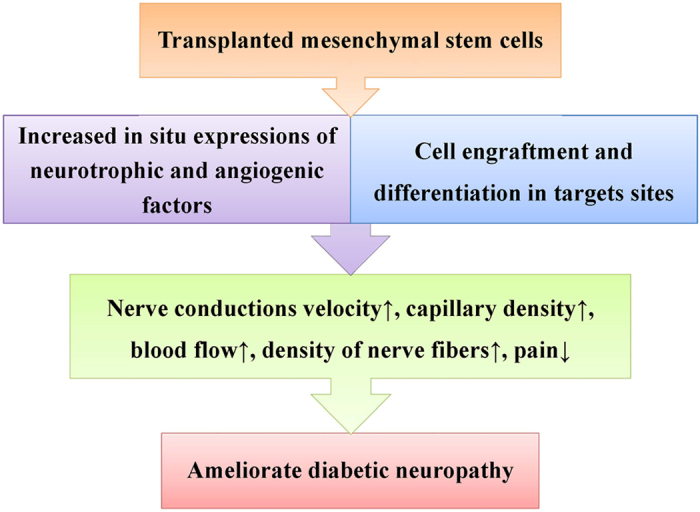
Mechanisms of the effect of stem cell transplantation on diabetic neuropathy.

MSC therapy may not be a standard treatment option for all stages of DN because different stages of DN are marked by different structural or functional changes. At present, MSC therapy may be applied to those patients who suffer from intractable symptoms, acute exacerbation, or combined diseases, such as diabetic foot ulcers or critical limb ischemia. MSC therapies targeting both vascular and neural elements are advantageous in treating DN.^50^ One recent meta-analysis shows that BMSC transplantation ameliorates allodynia but not hyperalgesia unless it is given during the first 4 days after injury.^51^ As shown in Figure 2, stem cells can improve DN through two main pathways.

### MSCs improve diabetic glycemic control

MSCs improve glycemic control, accompanied by improved renal function and regeneration of normal *β* pancreatic islets.^52^ Hypoglycemia of MSC transplantation is a direct effect of differentiation to cells capable of producing insulin (less likely) or an indirect effect of secretion of immunomodulators, which prevent T cells from eliciting pancreatic *β*-cell destruction, or other as yet unknown factors that influence insulin secretion or action. MSC differentiate into insulin-producing cells, releasing insulin in a glucose-dependant manner and improving diabetic symptoms in type 1 diabetic animal.^53,54^ These insulin-producing cells express multiple genes related to the development or function of pancreatic *β* cells.^54,55^ In diabetic NOD mice, the injection of MSC reduced the capacity of diabetogenic T cells to infiltrate pancreatic islets thus preventing *β*-cell destruction.^56^ An additional cooperative action of MSCs on co-transplantation with pancreatic islets results in improved graft morphology and improved revascularization, indicating that possible trophic factors secreted by MSCs are aiding islet engraftment.^57^ Multiple intravenous infusions of MSCs resulted in normalization of hyperglycemia, which remained stable for 9 weeks after infusion, with lower serum levels of insulin and C-peptide and reversed damaged pancreatic islets to near normal.^58^

### MSCs secret neurotrophic and angiogenic factors to ameliorate DN

MSCs offer a novel therapeutic option to treat DN. MSCs modulate the central nervous system-injured environment and promote repair as they secrete anti-inflammatory, antiapoptotic molecules, and trophic factors to support axonal growth, immunomodulation, angiogenesis, remyelination, and protection from apoptotic cell death.^59^ MSCs are known to support angiogenesis mostly through a paracrine effect, which augments the microcirculation supporting peripheral nerves. This impaired vascular supply has been implicated in the etiology of DN. Transplanted MSCs not only directly differentiate into neurons and endothelial cells but also secrete an increased concentrations of biologically active factors, such as FGF, VEGF-A, and NGF,^60,61^ which are central to nerve and vascular tissue health. Adipose-derived MSC sheet, which secret large amounts of several angiogenic growth factors *in vitro*, both directly and indirectly accelerate diabetic wound healing.^62^

BMSC transplantation increased the expression levels of FGF2 and VEGF, ameliorated sciatic nerve blood flow, prevented the decreases in the capillary-to-muscle ratio and the neurofilament content, and improved motor nerve conduction velocity in diabetic animals.^61,63^ Despite these benefits, however, motor nerve conduction velocity and the increase in the levels of NGF and neurotrophin 3 last for only 4 weeks.^64^ Interestingly, when it comes to neurotrophic factors, these two studies contradict each other. In one study,^64^ levels of NGF and neurotrophin 3, but not VEGF or FGF2, increase in the animals that received BMSC transplantation. In another study,^59^ however, VEGF and FGF2, but not NGF and neurotrophin 3, are found to increase in the animals that received stem cell transplantation. More studies are needed to understand the effects of MSCs on DN.

### MSCs inhibit proinflammation to improve diabetic peripheral neuropathy

The therapeutic benefit of MSCs in DN is now believed to be by short-term (hours to days) paracrine and juxtacrine modulation of immune responses rather than by long-term (days to months) engraftment of MSCs to the injured site.^10^ Subsequent improvements in MSC cell preparations to generate anti-inflammatory MSC populations resulted in improvements in behavioral assays in painful diabetic peripheral neuropathy, and mice treated with these MSCs showed decreased serum concentrations of proinflammatory cytokines.^10^ hMSCs reduce pain-like behaviors (mechanical allodynia and thermal hyperalgesia) after transplanted in cerebral ventricle. hMSCs have antinociceptive effect from day 10 after surgery (6 days after cell injection). hMSCs reduce the mRNA expression levels of interleukin-1*β* and neural *β*-galactosidase overactivation in prefrontal cortex of spared nerve injury mice.^65^ hMSCs reduce mechanical allodynia and thermal hyperalgesia via tail vein injection. An antinociceptive effect is measurable from day 11 after surgery (7 days after cell injection). hMSCs mostly home in the spinal cord and prefrontal cortex of neuropathic mice. Transplanted hMSCs downregulate the expression levels of the mouse interleukin-1*β* and interleukin-17 and upregulate the expression levels of interleukin-10 and the marker of alternatively activated macrophages CD106 in the spinal cord of spared nerve injury mice.^63^

### MSCs improve diabetic cardiac autonomic neuropathy

MSC administration promoted density of sympathetic and parasympathetic nerves in the ventricular myocardium of diabetic rats, increased the ratio of parasympathetic to sympathetic nerve fibers, and suppressed ventricular arrhythmia inducibility.^66^

### MSCs regenerate axons and format myelin to ameliorate DN

The injected BMSCs into hindlimb muscles of streptozotocin-induced diabetic rats restore motor and sensory nerve conduction velocities to near-normal levels. The injected MSCs are priority and durably engrafted in the sciatic nerves, and a fraction of the engrafted MSCs are discriminatively localized near to vasa nervora of sciatic nerves. MSCs increase the density of vasa nervora and restores the ultrastructure of myelinated fibers in nerves. MSCs also upregulate the gene expression of multiple factors participating in angiogenesis, neural function, and myelination in the MSC-injected nerves.^67^ The nerve grafts that are prepared from poly (3-hydroxybutyrate-co-3-hydroxyhexanoate) with oriented nanofiber three-dimensional surfaces aided to nerve regeneration, either used alone or with hMSC. Poly (3-hydroxybutyrate-co-3-hydroxyhexanoate) provided better nerve regeneration when used with hMSCs in combination than alone and reached the same statistical treatment effect in functional evaluation and electrophysiological evaluation when compared with autografting.^68^

## Challenge and future perspective

**Figure 3:**
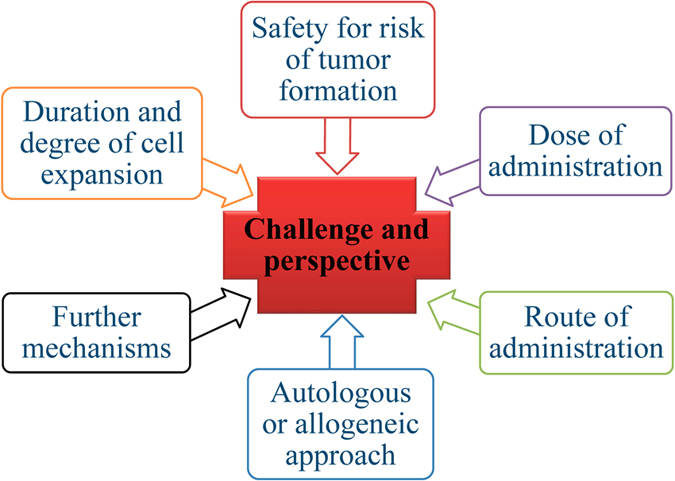
Challenges for clinical application of MSCs to treat diabetic neuropathy.

The application of the use of MSCs to treat DN has been extensively investigated in preclinical animal models in recent years and the majority of reports indicate positive effects on DN. Despite this, there are significant challenges to be met for the successful clinical translation of these studies from animal model to the patient’s bedside. Although MSC therapies protect from neurodegeneration and promote neuroregeneration, there appear to be many obstacles to be overcome for clinical applications (Figure 3). These are: (1) optimal dose of administration owing to limited survival of transplanted cells, (2) safety for risk of tumor formation, (3) route of transplantation for effectiveness, (4) autologous or allogeneic approach, impairing potency of MSCs from diabetes, (5) further mechanisms, and (6) clinical end points for the efficacy of MSC therapy.

### Safety

Even with the reported concerns over possible malignant transformation above, worldwide clinical studies of both autologous and allogeneic MSC administration have confirmed clinical safety and initial efficacy. A search of the ClinicalTrials.Gov website reveals that there are 612 open studies of MSC safety and efficacy in the treatment of human diseases by the year 2016. In relation to diabetes, there are 39 open clinical trials using MSCs to treat type 1 diabetes, type 2 diabetes, or their associated complications.

Another issue that should be overcome for the MSC therapy is to avoid the risk of tumor formation. Increased tumor formation was observed in animals owing to the immune-suppressive effect of MSCs especially with allogeneic transplants,^69^ and *in vitro* observations of sarcoma after culture of murine MSCs was reported.^70^ But one study indicated a tumor-suppressive activity of MSCs after preactivation with tumour necrosis factor-*α*.^71^ Frequent tumor formation in streptozotocin-induced diabetic mice transplanted with BMSCs was observed.^72^ The high frequency of tumor formation is accounted for by frequent chromosomal mutation elicited by repeated passages of BMSCs (only four passages) in the cell culture system. It may thus be conceivable that fresh BMSCs without any passage in cell culture should be applied to MSC therapy to prevent tumor formation.

### Dose of administration

The number of cells delivered are very important; however, there is still a lack of information as to the optimal cell doses that provide preclinical and clinical efficacy. One study demonstrated that hMSCs transplanted into animal model generated different grafts depending on the cell dosing: low numbers of transplanted hMSCs generated nestin-containing grafts, whereas higher numbers of transplanted hMSCs generated considerable amounts of grafts with astroglial markers.^42^ The number of cells transplanted also raises questions about cell survival; one study indicated that only 1.7% of total injected hMSCs survived.^48^ Despite all of their benefits, much research still needs to be carried out to understand the homing capabilities of stem cells^48^ and the mechanism of action.^14^ The optimal dose for stem cell transplantation needs further characterization prior to being introduced into clinical trials.

### Route of administration

Methods for transportation of MSCs without affecting their viability and efficacy are important along with issues related to cryopreservation. Several modes of cell delivery (e.g., topical, intraocular, and systemic) have been assessed in both preclinical and clinical studies, and these studies have illustrated the importance of administration route with the successful outcome. Systemic delivery is attractive as this may result in benefit for multiple complications and has the potential to improve glycemic control. Although an attractive option, the systemic delivery of MSCs has some barriers, such as homing of these cells to tissues of interest with high efficiency and clinically meaningful engraftment. More cells are required for injection owing to passive cell entrapment within non-specific tissues,^73,74^ and this can potentially lead to unwanted effects and reduced efficacy of transplanted cells. Topical application may be a very relevant alternate strategy for diabetic foot ulcers but this is approach can be limited by localized vascular damage as a result of diabetes at the site of administration. One approach is to implant cells repeatedly to maintain their effects. At present, the duration of the beneficial effects of MSC therapy in DN is unknown.

### Duration and degree of cell expansion

A major challenge is the large-scale production of MSCs under GMP conditions and issues of MSC heterogeneity. The duration and degree of cell expansion and culture has an impact on MSC morphology, differentiation, viability, and migratory properties. MSCs not only undergo phenotypic changes in culture and during passage (size, morphology, and cell surface marker expression)^75^ but also lose capacity for functional proliferation and differentiation potential.^75,76^ In addition, their ability for cytokine production is altered.^76^ Thus a delicate balance between culture expansion to gain sufficient numbers of MSCs for therapeutic application and long-term culture effects needs to be met. Tightly controlling the microenvironment of MSCs is required. Detailed investigations of how the microenvironment affects the immunosuppressive effects of MSCs are still lacking and are required as cell-to-cell contact and soluble factors are thought to be the key aspects of MSC-mediated immunosuppression.^37^

### Autologous or allogeneic approach

The choice of an autologous or allogeneic approach is an important consideration as the former may be limited by disease-induced cell dysfunction and the latter by an immune response to the transplanted cells. Historical opinions that the immunomodulatory functions of MSCs results in immune privilege for allogeneic MSC transplants are being challenged^77–79^ with the recommendation that the antidonor immune responses elicited by allogenic MSCs be studied in more detail. The limitation of allogenic MSC therapy may also be related to the gradual decrease in released neurotrophic factors from transplanted cells that may sustain only 4 weeks or so after transplantation.

### Mechanisms

Despite numerous studies on the transplantation of MSCs in animal models and patients, insight into the exact mechanisms of action underlying their beneficial effect remains unclear. Adequate preclinical animal models are required to accurately represent the pathological long-term effects of diabetes on the host system. There are limitations in the current rodent models of DN.^80^ There is an increased need for additional *in vitro* and *in vivo* studies to fully describe in detail the mechanisms of MSC therapy.

## Conclusion

DN frequently leads to foot ulcers and ultimately limb amputations without effective clinical therapy. DN is characterized by reduced vascularity in the peripheral nerves and deficiency in angiogenic and neurotrophic factors. Only delivering neurotrophic or angiogenic factors for treatment in the form of protein or gene therapy is very modest if not ineffective. MSCs have been highlighted as a new emerging regenerative therapy owing to their multipotency for DN. MSCs reverse manifestations of DN, repair tissue, and antihyperglycemia. MSCs also paracrinely secrete neurotrophic factors, angiogenic factors, cytokines, and immunomodulatory substances to ameliorate DN. Challenges in the clinical translation of MSC therapy include safety, optimal dose of administration, optimal mode of cell delivery, issues of MSC heterogeneity, clinically meaningful engraftment, autologous or allogeneic approach, challenges with cell manufacture, and further mechanisms.

## Abbreviations

BDNF: brain-derived nerve factor
BMSC: bone marrow-derived MSC
DN: diabetic neuropathy
FGF2: fibroblast growth factor-2
GDNF: glial cell line-derived neurotrophic factor
hMSC: human MSC
MSC: mesenchymal stem cell
NGF: nerve growth factor
VEGF: vascular endothelial growth factor.
